# Supplementary material for: Astrocytic GLUT1 deletion in adult mice enhances glucose metabolism and resilience to stroke
Source: Nat Commun. 2025 May 6;16:4190. doi: 10.1038/s41467-025-59400-2 (PMC12056070; doi:10.1038/s41467-025-59400-2)
Supplement: Supplementary file 1 — Supplementary Information [file 41467_2025_59400_MOESM1_ESM.pdf]

Supplementary Information

Astrocytic GLUT1 deletion in adult mice enhances glucose metabolism and resilience to stroke

Laetitia Thieren[1,2,#], Henri S. Zanker[1,2,#], Jeanne Droux[2,3], Urvashi Dalvi[1,2], Matthias T. Wyss[1,2], Rebecca Waag[2,4], Pierre-Luc Germain[2,4,5], Lukas M. von Ziegler[2,4], Zoe J. Looser[1,2], Ladina Hösli[1,2], Luca Ravotto[1,2], E. Dale Abel[6], Johannes Bohacek[2,4], Susanne Wegener[2,3], L. Felipe Barros[7,8], Mohamad El Amki[2,3], Bruno Weber[1,2*] and Aiman S. Saab[1,2*]

*[1] University of Zurich, Institute of Pharmacology and Toxicology, 8057, Zurich, Switzerland. [2] Neuroscience Center Zurich, University and ETH Zurich, 8057 Zurich, Switzerland. [3] Department of Neurology, University Hospital and University of Zurich, 8091 Zurich, Switzerland. [4] Lab of Molecular and Behavioral Neuroscience, Institute for Neuroscience, Department of Health Sciences and Technology, ETH Zurich, 8057, Zurich, Switzerland. [5] Lab of Statistical Bioinformatics, University of Zurich, 8057, Zurich, Switzerland. [6] Department of Medicine, David Geffen School of Medicine at UCLA, Los Angeles, CA 90095, USA. 7 Centro de Estudios Científicos (CECs), 5110465 Valdivia, Chile. [8] Facultad de Medicina y Ciencia, Universidad San Sebastián, 5110693, Valdivia, Chile. [#] These authors contributed equally: Laetitia Thieren, Henri S. Zanker. *correspondence: bweber@pharma.uzh.ch and asaab@pharma.uzh.ch*

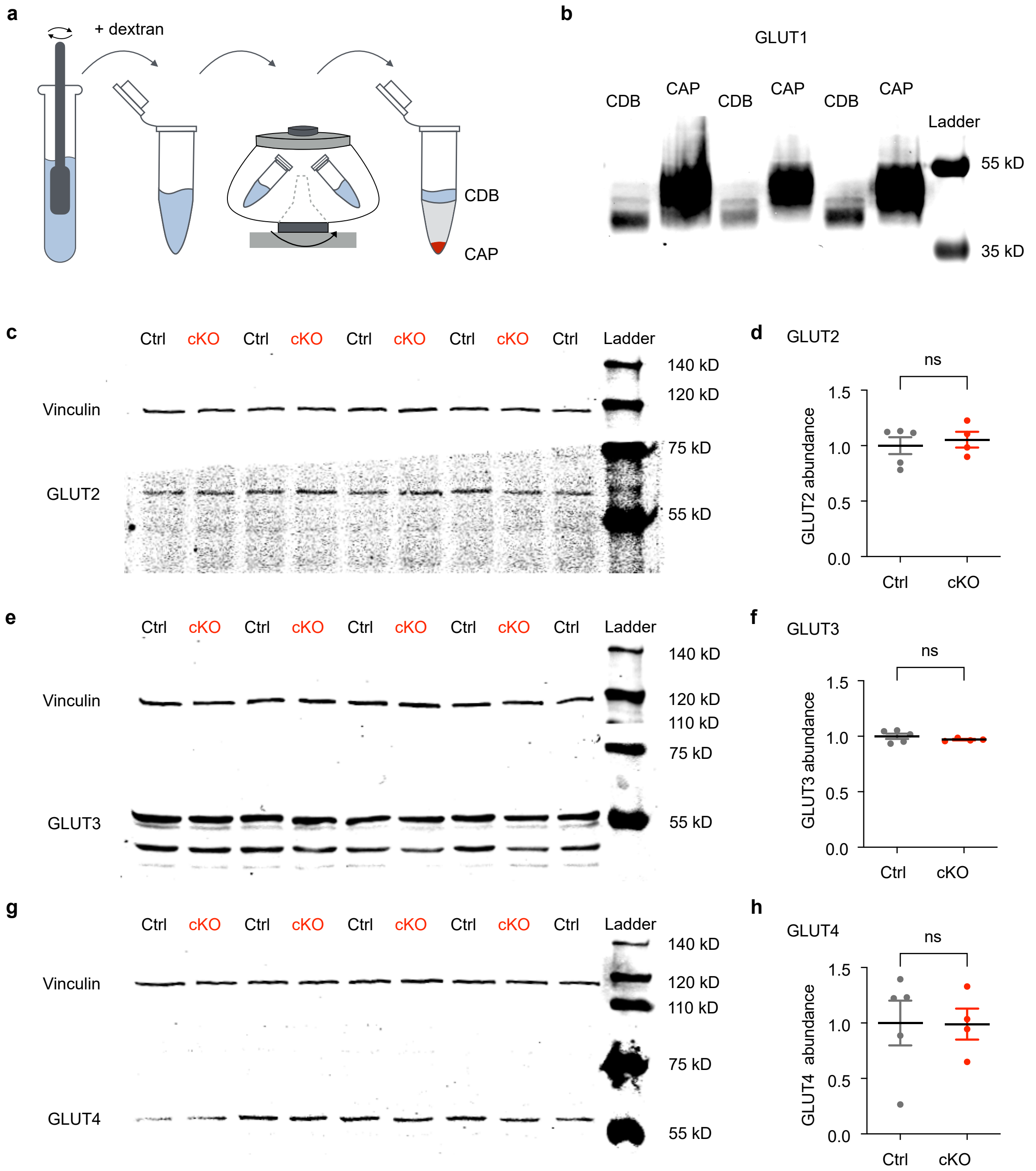

Supplementary Figure 1: Astrocytic GLUT1 deletion does not affect GLUT2, GLUT3, and GLUT4 abundance. **(a)** Scheme of the capillary-depleted brain (CDB) protocol. Forebrain tissue was homogenized, centrifuged, and the lysate was mixed with a 17% dextran solution. After centrifugation, the top layer contained CDB tissue, while capillaries (CAP) were pelleted at the bottom of the tube. **(b)** GLUT1 western blot showing a clear separation between CDB tissue enriched in astrocytic GLUT1 (~45 kDa) and CAP fractions containing endothelial GLUT1 (~55 kDa). **(c, d)** GLUT2 (~65 kDa) western blot analysis of CDB tissue shows similar abundance between GLUT1 cKO (n = 4) and Ctrl animals (n = 5, p = 0.6312, two-tailed unpaired t-test). Vinculin served as a loading control. **(e, f)** GLUT3 (~55 kDa) western blot analysis of CDB tissue shows similar abundance between GLUT1 cKO (n = 4) and Ctrl animals (n = 5, p = 0.3124, two-tailed unpaired t-test). Vinculin served as a loading control. **(g, h)** GLUT4 (~40 kDa) western blot analysis of CDB tissue shows similar abundance between GLUT1 cKO (n = 4) and Ctrl animals (n = 5, p = 0.9705, two-tailed unpaired t-test). Vinculin served as a loading control. Data are represented as a scatter dot plot with the mean ± SEM. Source data are provided as a Source Data file.

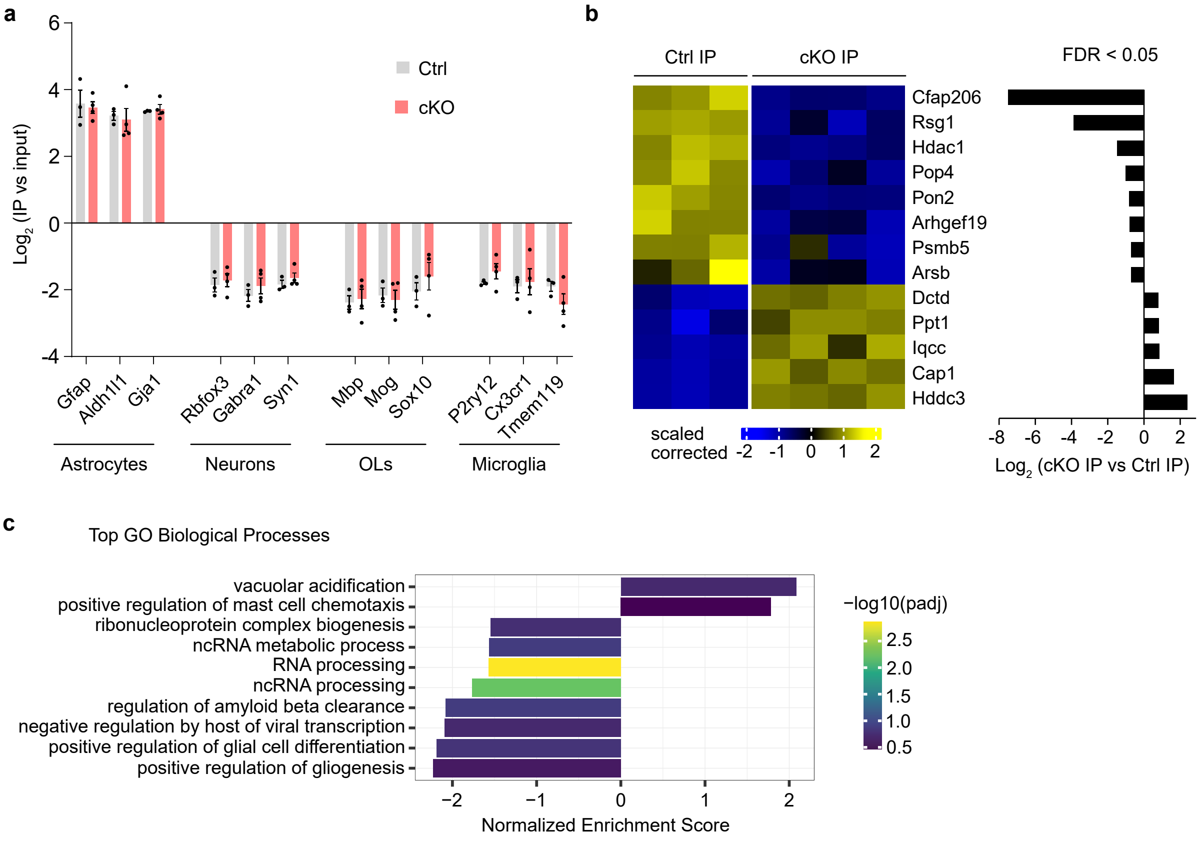

Supplementary Figure 2: Astrocytic translatome analysis in GLUT1 cKO mice. (a) Log2 fold change of expression of cell-specific markers between IP and input RNA from control (n = 3) and cKO (n = 4) mice . Note the enrichment of astrocyte-specific RNA in the IP samples of both genotypes. Data are represented as a scatter dot plot with the mean ± SEM. **(b)** Heatmap of all 13 differentially expressed genes (FDR < 0.05), ranked by log2 fold change between cKO IP and control IP. **(c)** Normalized enrichment scores of the top 10 gene ontology (GO) biological processes with significance indicated by -log10(padj) values. Source data are provided as a Source Data file.

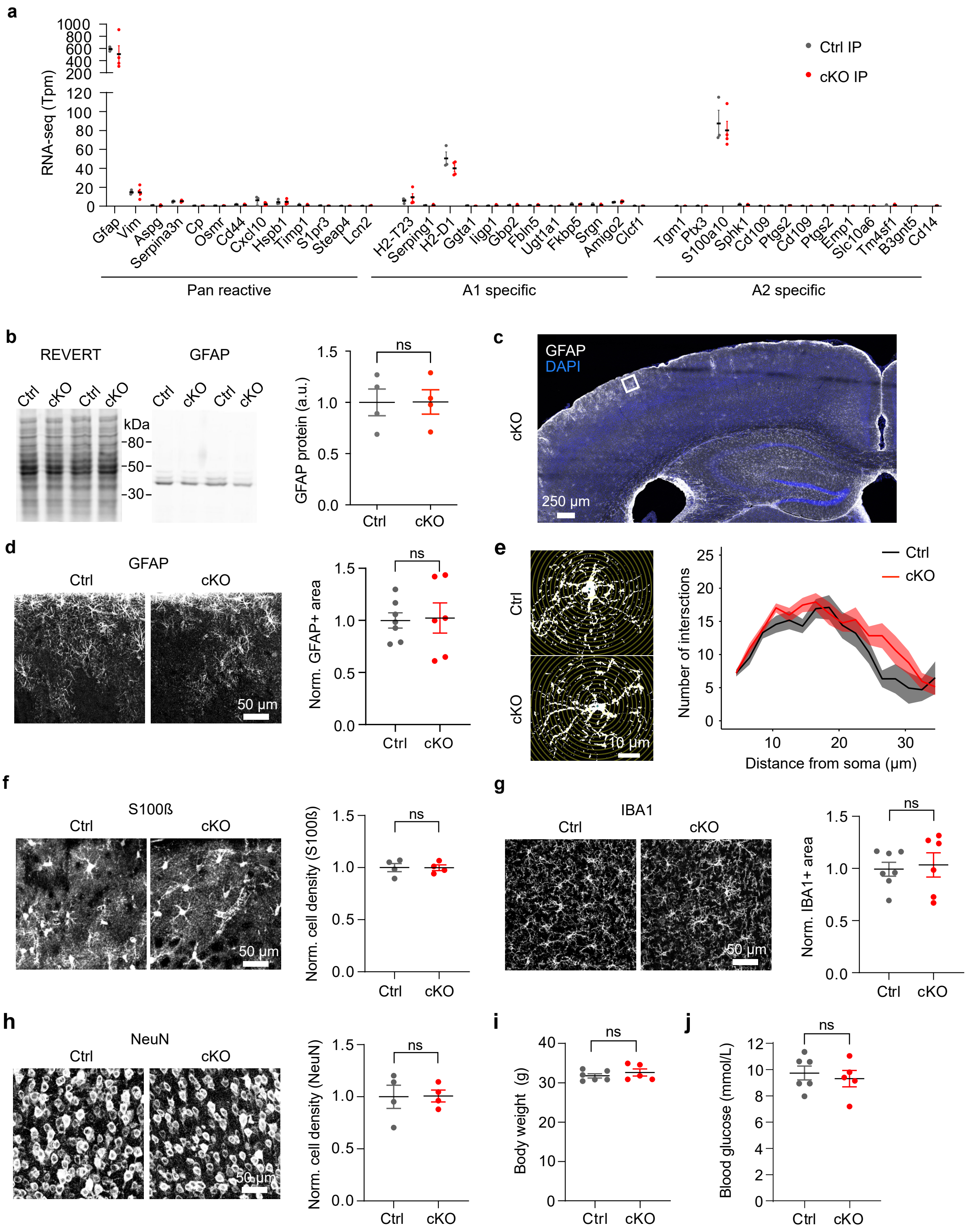

Supplementary Figure 3: No signs of gliosis or neurodegeneration in GLUT1 cKO mice. (a) No visible difference in expression levels (shown as transcripts per million, TPM) of actively translated mRNA related to pan-reactive, A1- and A2-specific astrocytes between Ctrl IP and cKO IP samples. **(b)** Western blot analysis of GFAP abundance in cortical extracts from Ctrl (n = 4) and cKO mice (n = 4) revealed no difference between genotypes (p = 0.9792, two-tailed unpaired t-test). **(c)** Overview image of GFAP and DAPI labeling of a cKO brain section, with a white square indicating the cortical region quantified in (d-h). **(d)** Confocal images and quantification of the GFAP-positive area in the cortex. No significant difference between genotypes (Ctrl n = 7, cKO n = 6, p = 0.9857, two-tailed unpaired t-test). **(e)** Sholl analysis revealed similar astrocyte morphology between genotypes. The line graph shows the quantification of the number of intersections (mean ± SEM) in Ctrl astrocytes (n = 10 cells, 3 animals) and cKO astrocytes (n = 17 cells, 4 animals). **(f)** Confocal images of the astrocytic marker S100β (left) and quantification of astrocytic density in the cortex (right) revealed no difference between genotypes (n = 4, p = 0.9857, two-tailed unpaired t-test). **(g)** Confocal images (left) and quantification (right) of the IBA1-positive area in the cortex. No significant difference between genotypes (Ctrl n = 7, cKO n = 6, p = 0.7294, two-tailed unpaired t-test). **(h)** Confocal images of the neuronal marker NeuN (left) and quantification of neuronal density in the cortex (right) revealed no difference between genotypes (n = 4, p = 0.9553, two-tailed unpaired t-test). **(i)** Similar body weight between genotypes (n = 6 vs. 5 male littermates, p = 0.4021, two-tailed unpaired t-test). **(j)** Similar blood glucose levels between genotypes (n = 6 vs. 5 male littermates, p = 0.6166, two-tailed unpaired t-test). Data are represented as the mean ± SEM. Source data are provided as a Source Data file.

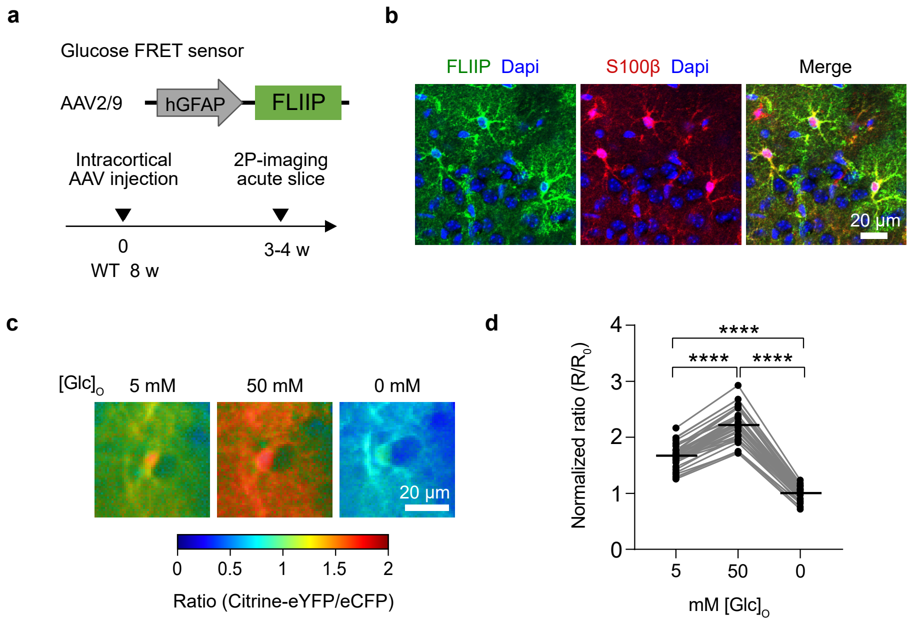

Supplementary Figure 4: Glucose sensor imaging in astrocytes in acute brain slices. (a) Intracortical AAV-mediated expression of glucose FRET sensor FLII12Pglu700µΔ6 (termed FLIIP) in 8-week-old wild-type mice. **(b)** Immunohistochemistry for S100β confirms astrocyte-specific expression of FLIIP (anti-GFP) 3 weeks after AAV delivery. **(c)** Representative color-coded ratio images from cortical astrocytes in ACSF containing 5 mM glucose ($[Glc]_O$), after elevating $[Glc]_O$ to 50 mM and after removal of extracellular Glc. Warm and cold colors indicate high and low glucose levels (or ratios), respectively. **(d)** Quantification of normalized ratios obtained from conditions presented in (c). Ratios of individual astrocytes (n = 36 cells obtained from 4 brain slices of 2 WT mice) were normalized to the averaged minimum obtained at 0 mM $[Glc]_O$. Glucose levels significantly increased in 50 mM $[Glc]_O$ and decreases 0 mM $[Glc]_O$ ($p < 0.0001$, one-way Anova with Tukey's multiple comparisons test). Data shown as scatter dot plot and the mean. Source data are provided as a Source Data file.

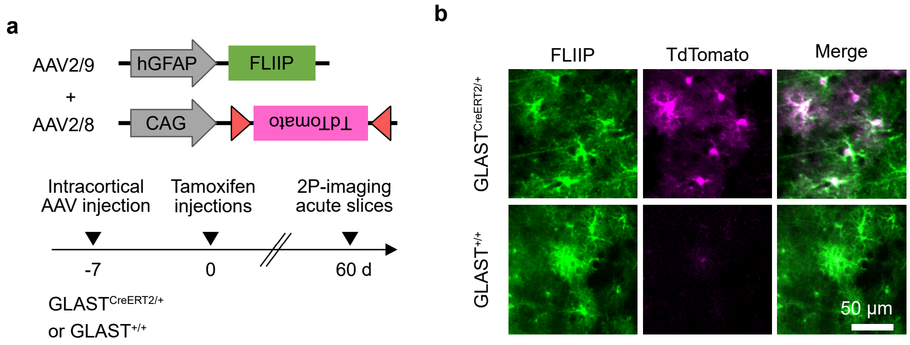

Supplementary Figure 5: Validation of Cre-reporter AAV encoding TdTomato. (a) At 8 weeks of age, GLAST$^{CreERT2/+}$ and GLAST$^{+/+}$ animals were injected with AAVs encoding FLIIP together with the Cre-reporter AAV (DIO-TdTomato). They were treated with tamoxifen 7 days post-AAV injection for acute cortical slice two-photon (2P) imaging 60 days after tamoxifen treatment. **(b)** Example 2P images obtained from cortical slices showing astrocytes co-expressing TdTomato (indicating cells targeted for Cre recombination) exclusively in mice expressing CreERT2, confirming the specificity of the Cre-reporter AAV. Excitation wavelengths of 870 and 1050 nm were used to reliably detect FLIIP and TdTomato, respectively. Observed in three mice per genotype.

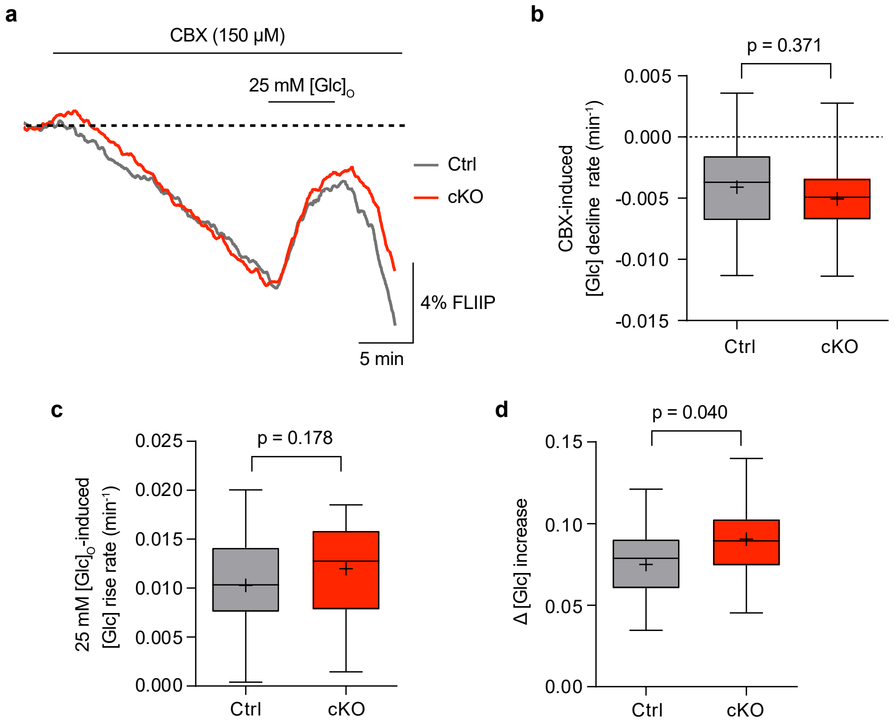

Supplementary Figure 6: Glucose uptake in GLUT1 cKO astrocytes is independent of gap junction coupling. **(a)** Representative traces of astrocytic glucose dynamics during Carbenoxolone (CBX) incubation and upon transient elevation of $[Glc]_O$ from 5 mM to 25 mM in Ctrl and cKO brain slices. **(b)** CBX-induced glucose decline rate was comparable between cKO astrocytes (n = 58 cells; from 8 brain slices, 4 animals) and Ctrl astrocytes (n = 53 cells; from 8 brain slices, 4 animals; p = 0.371, linear mixed model analysis). **(c)** Transient 25 mM $[Glc]_O$-induced glucose uptake rate was similar between the genotypes (p = 0.178, linear mixed model analysis). **(d)** The glucose rise (Δ[Glc] increase) in the presence of CBX was significantly higher in cKO astrocytes compared to Ctrl (p = 0.040, linear mixed model analysis). Box plots show the median (center line), quartiles (box bounds), mean (+) and min-max (whiskers). Source data are provided as a Source Data file.

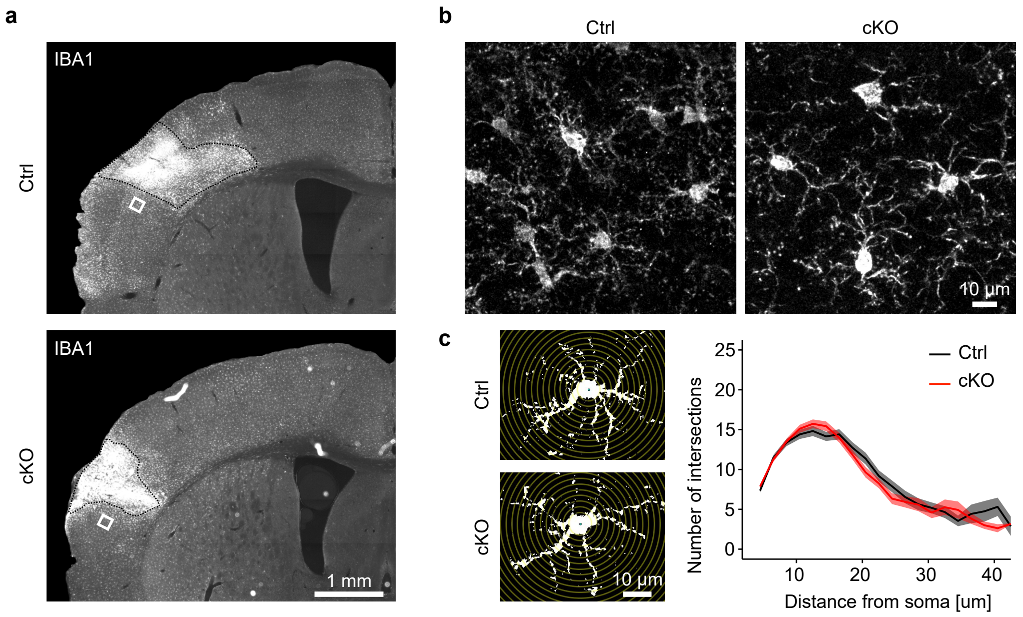

Supplementary Figure 7: No difference in peri-infarct microglia morphology between genotypes. (a) Representative images of brain sections from Ctrl and cKO mice 7 days post-stroke, stained with IBA1, a marker for microglia. The dashed outline demarcates the lesion area, and the white square indicates the region analyzed in (b, c). Note the intense IBA1 labeling in the infarct area. **(b)** Representative images of microglia in the peri-infarct region, indicated by the white square in (a). **(c)** Sholl analysis of peri-infarct microglia (see also Fig. 5g). The line graph shows the quantification of the number of intersections. Peri-infarct microglia from Ctrl ($n = 77$ cells from 8 animals) and cKO mice ($n = 92$ cells from 8 animals) show no significant difference in the number of intersections at any distance from the soma ($p > 0.17$, linear mixed-effects model with post hoc pairwise comparisons). Data are presented as mean ± SEM. Source data are provided as a Source Data file.

Uncropped scans:

Suppl. Fig. 1 c:

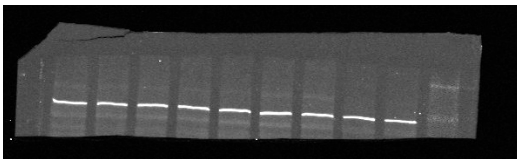

Vinculin

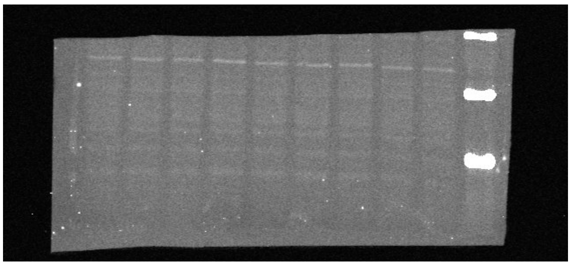

GLUT2

Suppl. Fig. 1 d:

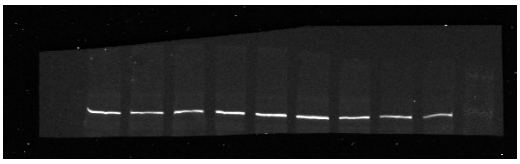

Vinculin

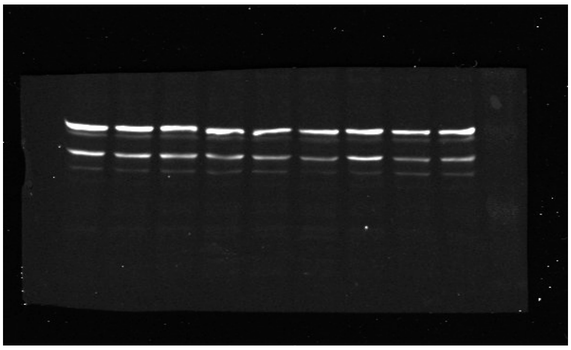

GLUT3

Suppl. Fig. 1 g:

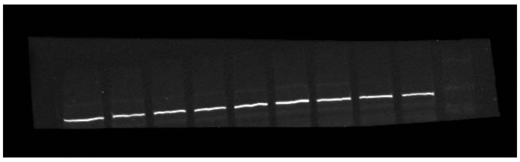

Vinculin

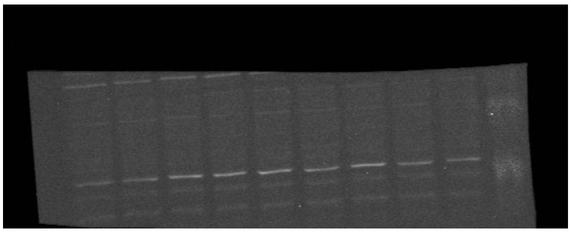

GLUT4

Suppl. Fig. 2 b:

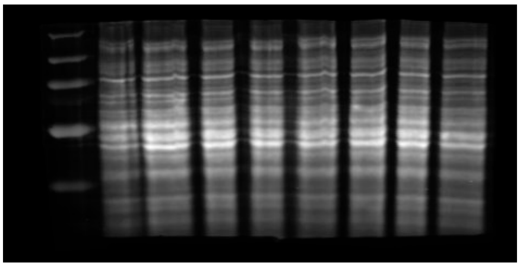

REVERT Total Protein Stain

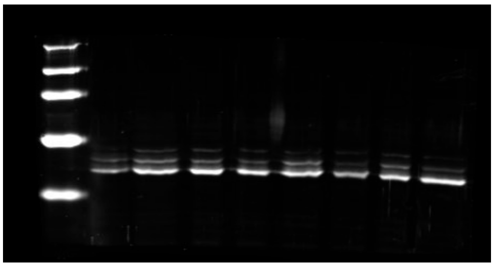

GFAP
