# Supplementary material for: Astrocytic GLUT1 deletion in adult mice enhances glucose metabolism and resilience to stroke
Source: Nat Commun. 2025 May 6;16:4190. doi: 10.1038/s41467-025-59400-2 (PMC12056070; doi:10.1038/s41467-025-59400-2)
Supplement: Supplementary file 2 — Reporting Summary [file 41467_2025_59400_MOESM2_ESM.pdf]

nature portfolio

Corresponding author(s): Aiman S. Saab

Last updated by author(s): Apr 6, 2025

Reporting Summary

Nature Portfolio wishes to improve the reproducibility of the work that we publish. This form provides structure for consistency and transparency in reporting. For further information on Nature Portfolio policies, see our Editorial Policies and the Editorial Policy Checklist.

Statistics

For all statistical analyses, confirm that the following items are present in the figure legend, table legend, main text, or Methods section.

n/a	Confirmed	
☐	☒	The exact sample size (*n*) for each experimental group/condition, given as a discrete number and unit of measurement
☐	☒	A statement on whether measurements were taken from distinct samples or whether the same sample was measured repeatedly
☐	☒	The statistical test(s) used AND whether they are one- or two-sided<br>*Only common tests should be described solely by name; describe more complex techniques in the Methods section.*
☐	☒	A description of all covariates tested
☒	☐	A description of any assumptions or corrections, such as tests of normality and adjustment for multiple comparisons
☐	☒	A full description of the statistical parameters including central tendency (e.g. means) or other basic estimates (e.g. regression coefficient) AND variation (e.g. standard deviation) or associated estimates of uncertainty (e.g. confidence intervals)
☐	☒	For null hypothesis testing, the test statistic (e.g. *F*, *t*, *r*) with confidence intervals, effect sizes, degrees of freedom and *P* value noted<br>*Give P values as exact values whenever suitable.*
☒	☐	For Bayesian analysis, information on the choice of priors and Markov chain Monte Carlo settings
☒	☐	For hierarchical and complex designs, identification of the appropriate level for tests and full reporting of outcomes
☒	☐	Estimates of effect sizes (e.g. Cohen's *d*, Pearson's *r*), indicating how they were calculated

Our web collection on statistics for biologists contains articles on many of the points above.

Software and code

Policy information about availability of computer code

Data collection	Immunohistochemistry: All images were acquired using a Zeiss LSM 700 or Zeiss LSM 800 confocal laser scanning microscope mounted with a 10x (Plan-Apochromat, NA 0.45), a 25x (LCI Plan-Neofluar, NA 0.8) or a 40x objective (Plan-Apochromat, NA 1.4, Oil DIC (UV) VIS-IR). Whole brain section pictures were taken with the 10X and tiled images were aligned with the built-in microscope software (ZEN software, Zeiss).<br>For RNA-sequencing analysis, the cDNA and generation of libraries were performed with the Smart-seq2 protocol (Picelli et al., 2014). Single-end sequencing (100nt) was performed at the Functional Genomics Center Zurich (FGCZ) core facility with an Illumina Novaseq 6000.<br>Two-photon imaging: custom-built two-photon microscope (Mayrhofer et al., 2015), equipped with a tunable pulsed Ti:Sapphire laser (Chameleon Ultra II; Coherent) and a 20x water immersion objective (W Plan-Apochromat 20x/1.0 DIC VIS-IR, Zeiss). The microscope was controlled by a customized version of ScanImage (r3.8.1; Janelia Research Campus, (Pologruto et al., 2003)). Fluorescence emission was detected with a GaAsP photomultiplier tube (PMT; Hamamatsu Photonics) using band-pass filter 520/70 nm (Semrock) or a dichroic beam-splitter (560 nm edge, BrightLine; Semrock) and two band-pass filters 545/55 nm and 475/50 nm (Semrock).<br>Cerebral flood flow (CBF) imaging: CBF changes were monitored before and during ischemia, using a laser speckle contrast imaging monitor (FLPI, Moor Instruments, UK). The acquisition was performed with a frame rate of 0.25 Hz. LSI images were generated with arbitrary units in a 256-colour palette by the MOOR-FLPI software.<br>Behavioral data: Behavioral videos were acquired using a GoPro camera (HERO4).
Data analysis	MATLAB (MathWorks, R2015b), ImageJ (Fiji version 1.52p), GraphPad Prism 9, R (v.3.2.2, R Core Team, 2015).<br>RNA-sequencing analysis: The coverage of Slc2a1 exons was based on read counts in a STAR alignment; for all other purposes kallisto (Bray et al., 2016) was used for pseudo alignment of reads on the transcriptome level using the genecode.vM17 assembly with 30 bootstrap samples and an estimated fragment length of 200±20. One sample was excluded due to a very low read count, and IP samples that showed a high expression of neuronal genes were excluded. For differential gene expression (DGE) analysis we aggregated reads of protein coding transcripts and used R (v. 3.6.2) with the package "edgeR" (v 3.26.8) for analysis. A filter was used to remove genes with low expression prior

to DGE analysis. edgeR was then used to calculate the normalization factors (TMM method) and estimate the dispersion (by weighted likelihood empirical Bayes). For two group comparisons the genewise exact test was used. For multiple testing correction the Benjamini–Hochberg false discovery rate (FDR) method was used. GO enrichment analysis was done with topGO, using Fisher's test and the weight01 algorithm. Heatmaps were produced with the sechm package. To avoid rare extreme values from driving the scale, the color scale is linear for values within a 98% interval, and ordinal for values outside it. Unless otherwise specified, the rows were sorted using the features' angle on a two-dimensional projection of the plotted values, as implemented in sechm.
Behavioral analysis: Video recordings were analyzed using DeepLabCut (2.1.8.1) and R (3.6.1). In brief, we trained a model to track nose, ears and tailbase of the mouse for each frame of the video. These markers were mapped against ROIs for each of the holes in R which allowed counting the number of nose-pokes into each of the holes as well as measuring latency and distance walked.
Metabolic imaging: Code used for FRET image analysis is available at GitHub (https://gitlab.com/einlabzurich/fretanalysis).

For manuscripts utilizing custom algorithms or software that are central to the research but not yet described in published literature, software must be made available to editors and reviewers. We strongly encourage code deposition in a community repository (e.g. GitHub). See the Nature Portfolio guidelines for submitting code & software for further information.

Data

Policy information about availability of data

All manuscripts must include a data availability statement. This statement should provide the following information, where applicable:

- Accession codes, unique identifiers, or web links for publicly available datasets
- A description of any restrictions on data availability
- For clinical datasets or third party data, please ensure that the statement adheres to our policy

The RNA-sequencing data reported in this study have been deposited at Gene Expression Omnibus (GEO) under the accession code GSE223687. All other data are available from the corresponding author upon request. Source data are provided with this paper.

Research involving human participants, their data, or biological material

Policy information about studies with human participants or human data. See also policy information about sex, gender (identity/presentation), and sexual orientation and race, ethnicity and racism.

Reporting on sex and gender	N/A
Reporting on race, ethnicity, or other socially relevant groupings	N/A
Population characteristics	N/A
Recruitment	N/A
Ethics oversight	N/A

Note that full information on the approval of the study protocol must also be provided in the manuscript.

Field-specific reporting

Please select the one below that is the best fit for your research. If you are not sure, read the appropriate sections before making your selection.

☒ Life sciences ☐ Behavioural & social sciences ☐ Ecological, evolutionary & environmental sciences

For a reference copy of the document with all sections, see nature.com/documents/nr-reporting-summary-flat.pdf

Life sciences study design

All studies must disclose on these points even when the disclosure is negative.

Sample size	The provision of age-matched transgenic mouse cohorts limited the ability to determine sample sizes in advance. No statistical methods were used to predetermine sample size. Sample sizes used in this study are equivalent to the standard in the field.
Data exclusions	Sometimes sensor expression was too weak (e.g. AAV delivery failure), these animals were excluded from experiments.
Replication	Number of repetitions (individual data points from each cells and/or animal) are indicated in figures or figure legends.
Randomization	Selection of mice was based on genotype or wildtype mice were ordered from Charles River. Mice were randomly assigned to experimental groups, depending on availability of transgenic cohorts.
Blinding	Experimenters were blinded for the genotypes. And whenever possible, investigators were blinded for data analysis.

Reporting for specific materials, systems and methods

We require information from authors about some types of materials, experimental systems and methods used in many studies. Here, indicate whether each material, system or method listed is relevant to your study. If you are not sure if a list item applies to your research, read the appropriate section before selecting a response.

Materials & experimental systems

n/a	Involved in the study
☐	☒ Antibodies
☒	☐ Eukaryotic cell lines
☒	☐ Palaeontology and archaeology
☐	☒ Animals and other organisms
☒	☐ Clinical data
☒	☐ Dual use research of concern
☒	☐ Plants

Methods

n/a	Involved in the study
☒	☐ ChIP-seq
☒	☐ Flow cytometry
☒	☐ MRI-based neuroimaging

Antibodies

Antibodies used

Antibody information. IHC, immunohistochemistry, WB, Western blot
Rabbit polyclonal anti-GLUT1 (IHC 1:300, WB 1:15000); prod. by Kathrin Kusch (Berghoff et al., 2017): antibody produced by Kathrin Kusch from the Department of Neurogenetics, Max Planck Institute for Multidisciplinary Sciences, Göttingen, Germany.
Rabbit polyclonal anti-GLUT2 (WB) 1:15000 Abcam Cat# ab54460
Rabbit monoclonal anti-GLUT3 (WB) 1:15000 Abcam Cat# ab191071
Rabbit polyclonal anti-GLUT4 (WB) 1:15'000 Millipore Cat# 07-1404
Mouse monoclonal anti-GS (IHC) 1:700 BD Transduction Laboratories Cat# 610518
Chicken polyclonal anti-GFAP (IHC) 1:2000 Abcam Cat# Ab4674
Rabbit polyclonal anti-GFAP (IHC) 1:1'000 DAKO Cat# Z334
Rabbit polyclonal anti-Iba1 (IHC) 1:1000 FUJIFILM Wako Chemicals Cat# 019-19741
Goat polyclonal anti-IBA1 (IHC) 1:1000 Abcam Cat# ab5076
Rabbit monoclonal anti-S100β (IHC) 1:700 Abcam Cat# ab52642
Rabbit monoclonal anti-NeuN (IHC) 1:1500 Abcam Cat# ab177487

Validation

Quality control information and relevant citations are available at manufacturer's website.
anti-GLUT1: (Berghoff et al., 2017; Looser at al., 2024)
anti-GLUT2: https://www.abcam.com/en-ch/products/primary-antibodies/glucose-transporter-glut2-antibody-ab54460#application=wb
anti-GLUT3: https://www.abcam.com/en-ch/products/primary-antibodies/glucose-transporter-glut3-glut14-antibody-epr10508n-n-terminal-ab191071#application=wb
anti-GLUT4: https://www.merckmillipore.com/CH/de/product/Anti-GLUT-4-Antibody-C-terminus,MM_NF-07-1404?ReferrerURL=https%3A%2F%2Fwww.google.com%2F
anti-GS: https://www.bdbiosciences.com/en-ch/products/reagents/microscopy-imaging-reagents/immunofluorescence-reagents/purified-mouse-anti-glutamine-synthetase.610518
anti-GFAP abcam: https://www.abcam.com/en-gb/products/primary-antibodies/gfap-antibody-ab4674
anti-GFAP Dako: https://www.citeab.com/antibodies/2452274-z0334-glial-fibrillary-acidic-protein-gfap
anti-IBA1: https://labchem-wako.fujifilm.com/us/product/detail/W01W0101-1974.html
anti-IBA1: https://www.abcam.com/en-us/products/primary-antibodies/iba1-antibody-ab5076?srsltid=AfmBOoq9lvhL8MsBlCR9xTGa8wx3Qqq76KLzBh94zsD3i9DBrQrMybBY#tab=images
anit-S100β: https://www.abcam.com/en-gb/products/primary-antibodies/s100-beta-antibody-ep1576y-astrocyte-marker-ab52642
anti-NeuN: https://www.abcam.com/en-gb/products/primary-antibodies/neun-antibody-epr12763-neuronal-marker-ab177487

Animals and other research organisms

Policy information about studies involving animals; ARRIVE guidelines recommended for reporting animal research, and Sex and Gender in Research

Laboratory animals

Mice carrying the floxed Slc2a1 allele (GLUT1fl/fl, Fidler et al., 2017) were crossbred with GLASTCreERT2/+ (Mori et al., 2006) mice to obtain GLUT1fl/fl;GLASTCreERT2/+ mice. Control animals were either GLUT1fl/fl;GLAST+/+ or GLUT1+/+;GLASTCreERT2/+ mice. For experiments in wild-type animals, Charles River C57BL/6J mice (2-3 months old) were used. Mice targeted for recombination were injected with Tamoxifen at 8–10 weeks of age, and experiments were conducted from 60 to 90 days post-injection.

Wild animals

no wild animals were used in the study

Reporting on sex

For behavioral and stroke-related experiments, only male mice were used to minimize variability associated with sex differences in stroke outcomes and behavior. For all other analyses, both sexes were included whenever possible, based on cohort availability. This study was not designed to investigate sex-specific effects; animals were used as available, and post hoc sex-based analyses were not performed due to insufficient sample sizes for meaningful statistical conclusions.

Field-collected samples	no field collected samples were used in the study
Ethics oversight	All animal experiments were permitted by the local veterinary authorities in Zurich, in agreement with the guidelines of Swiss Animal Protection Law, Veterinary Office, Canton Zurich (Animal Welfare Act of 16 December 2005 and Animal Welfare Ordinance of 23 April 2008).

Note that full information on the approval of the study protocol must also be provided in the manuscript.

Plants

Seed stocks	N/A
Novel plant genotypes	N/A
Authentication	N/A
